# Proteases from* Canavalia ensiformis*: Active and Thermostable Enzymes with Potential of Application in Biotechnology

**DOI:** 10.1155/2016/3427098

**Published:** 2016-08-17

**Authors:** Rayane Natshe Gonçalves, Suellen Duarte Gozzini Barbosa, Raquel Elisa da Silva-López

**Affiliations:** Department of Natural Products, Farmanguinhos, Oswaldo Cruz Institute (FIOCRUZ), Avenida Brasil 4365, 21045-900 Rio de Janeiro, RJ, Brazil

## Abstract

Extracts of leaves, seeds, roots, and stem from a tropical legume,* C. ensiformis*, were prepared employing buffers and detergent in aqueous solution. Leaf extracts had the highest protein content and the most pronounced peptidase activity with optimal pH in the neutral to alkaline range. All extracts exhibited peaks of activity at various pH values, suggesting the presence of distinctive classes of proteases. N-*α*-Tosyl-L-arginine methyl ester hydrolysis was maximal at 30°C to 60°C and peptidase activity from all extracts presented very good thermal stability after 24 h incubation at 70°C.* C. ensiformis* proteases exhibited molecular masses of about 200–57, 40–37, and 20–15 kDa by SDS-PAGE analysis. These enzymes cleaved hemoglobin, bovine serum albumin, casein, and gelatin at different levels. Serine and metalloproteases are the major proteases in* C. ensiformis* extracts, modulated by divalent cations, stable at 1% of surfactant Triton X-100 and at different concentrations of the reducing agent *β*-mercaptoethanol. Thus,* C. ensiformis* expresses a particular set of proteases in distinctive organs with high activity and stability, making this legume an important source of proteases with biotechnological potential.

## 1. Introduction 

Proteases, also known as peptidases, are found in all living organisms and are able to hydrolyze the peptide bounds in proteins and peptides in different environments under many conditions. Thus, there are distinctive types of proteolytic enzymes that may be classified as exopeptidases, which act on the ends of protein substrates and endopeptidases acting on the interior of protein substrates. Further subclassification is based on the type of functional group at the active site. The hydroxyl group of serine proteases (EC 3.4.21) and threonine proteases (EC 3.4.25) and the sulfhydryl group of cysteine proteases (EC 3.4.22) are the nucleophile in catalysis, while activated water is the nucleophile for aspartic proteases (EC 3.4.23), glutamic proteases (EC 3.4.19), and metalloproteases (EC 3.4.24). Proteases are encoded by approximately 2% of all genes of an organism and plant genomes encode hundreds of proteases, which represent dozens of unrelated families and are responsible for protein metabolism [1].

Plant proteolysis is not limited to protein turnover to provide amino acids, carbon, and nitrogen for the synthesis of new molecules [2]. However, the proteases are important mediators of a striking variety of biological processes, since they cleave specific peptide bonds in key proteins and enzymes, involving irreversible reactions, and thus are involved in the regulation of growth and development [3]. They are involved in gene expression control that is responsible for many physiological processes such as cell growth, differentiation, division, and reproduction, as well as senescence, meiosis, gametophyte survival, epidermal cell fate, stomata development, chloroplast biogenesis, removal of damaged or improperly folded proteins, processing and targeting of proteins, and activation of zymogens and peptide hormones through digestion of signal peptides by limited cleavages; they participate in apoptosis and control metabolism and organ development and local and systemic defense responses [3–7]. These enzymes accumulate in different subcellular compartments [8].

*Canavalia ensiformis* (L.) DC or jack bean is a tropical legume, a vigorous herbaceous annual climber or woody shrub, native to Central America and cultivated worldwide [9]. It is resistant to insects and microorganisms and suppresses the growth of nematodes [10, 11]. In agriculture,* C. ensiformis* is used as a green cover for the nutritional enrichment of soils, because it fixes nitrogen efficiently. Due to the high nutritional values, it serves for human and cattle consumption after cooking to inactivate the toxins [3]. The seeds are rich sources of proteins with biotechnological importance including ureases [4, 5], proteases [6–8], and the lectin concanavalin A [9]. Species of the Fabaceae family, such as* C. ensiformis*, accumulate and mobilize large amounts of proteins in their seeds as a consequence of their extensive protein metabolism, which is more intense than that of plants from other botanic families [3]. In order to regulate both protein and general metabolisms, legumes express high levels of proteases and their specific inhibitors [12, 13]. It is important to point out that proteases constitute a very significant group of industrial enzymes with annual sales of about $1.5–1.8 billion and account for 60% of the total enzyme market [14]. Plant proteases, like papain, bromelain, and ficin, are widely used as therapeutic enzymes in wound healing and in the treatment of cancer, digestion disorders, and infectious diseases, as well as in various processes of food industry such as brewing, meat tenderization, and milk curdling. These plant enzymes have the advantages of high activity, ready availability, and low cost of production [15]. Thus, the aim of the present study was to identify protease activities in aqueous extracts from different parts of* C. ensiformis* with high proteolytic activity and good stability towards heat and chemical agents, desirable properties for biotechnological application.

## 2. Materials and Methods 

### 2.1. Plant

*Canavalia ensiformis* (L.) DC parts were collected in the morning on sunny days, in different seasons of the year, from the Atlantic Forest campus of the Oswaldo Cruz Foundation (FIOCRUZ) in the state of Rio de Janeiro, Brazil (S: 22° 56′ 24.10′′/W: 43° 24′ 09.22′′). The plant specimen was deposited in the Rio de Janeiro Botanical Garden, Rio de Janeiro, Brazil, under number RB-550.352.

### 2.2. Preparation of Extracts

Fresh leaves (about 300 g for each extraction) were powdered using liquid nitrogen, and proteins were extracted using water, buffers, or aqueous solution of detergent for 2 h at room temperature (24°C), with gentle stirring followed by centrifugation at 10,000 ×g for 30 min at 4°C. The supernatants were collected and lyophilized giving four leaf extracts: an aqueous extract (CE-A) using only distilled water; a detergent extract (CE-D) using 1% Triton X-100; a phosphate extract (CE-P) using 50 mM sodium phosphate with pH 6.5; and a Tris extract (CE-T) using 50 mM Tris-HCl pH 7.5. Fresh seeds (about 250 g), stems (about 150 g), and roots (about 85 g) were homogenized in distilled water using a blender, and the supernatants obtained after centrifugation were lyophilized to give the aqueous extracts from seeds (CE-SA), stem (CE-CA), and root (CE-RA), respectively. The protein content was measured by the method of Bradford [16] in order to minimize the interference of plant alkaloids and polyphenols, using BSA as a standard.

### 2.3. Determination of Optimal pH, Temperature, and Heat Stability

The assays for pH dependence were carried out by incubating 10 *µ*g of protein from the* C. ensiformis* extracts for 15 min at room temperature with 0.125 mM N-*α*-tosyl-L-arginine methyl ester (L-TAME) using different buffers. L-TAME is a substrate that contains arginine at the P1 site and is suitable for serine proteases such as trypsin, thrombin, and plasmin; however, it can be hydrolyzed, less specifically, by other protease classes [17]. The buffers, used at 50 mM, were as follows: sodium citrate (pH 4.0–6.5) or Tris-HCl (pH 7.0–8.5) or sodium carbonate/bicarbonate (pH 9.0–10.0). To determine the optimum temperature of enzyme activity, L-TAME and 10 *µ*g of protein from the extracts were incubated with the selected 50 mM buffer for 15 min at different temperatures ranging from 20°C to 60°C. Absorbance was monitored at 247 nm and each assay was performed in triplicate. Specific activity was expressed in *µ*mol min^−1^ mg^−1^ of protein. For thermal stability assays, extracts were incubated prior to the assay with selected buffers at 70°C for up to 24 h. The reaction was triggered by adding L-TAME at room temperature. The residual activity was calculated considering the protease activity (at 24°C) without previous incubation as 100%. The results of each series were expressed as the mean value ± SD.

### 2.4. Polyacrylamide Gel Electrophoresis and Substrate Gel Electrophoresis

Protein profiles of extracts were analyzed by sodium dodecyl sulfate polyacrylamide gel electrophoresis (SDS-PAGE) according to the method of Laemmli [18], and the gels were stained with Coomassie Blue R-250. For molecular mass characterization, Precision Plus Protein Standards (250–10 kDa) from Bio-Rad (Berkeley, CA, USA) were used as molecular standards. Enzymatic activity of extracts was first analyzed by gelatin substrate gel electrophoresis and was carried out under reducing conditions as previously reported [19]. After electrophoresis, the gel was washed with 2.5% Triton X-100 for 1 h to remove SDS and incubated overnight at room temperature in different 50 mM buffers to allow proteolysis. The next day, gels were stained with 0.1% amide black and destained in methanol/acetic acid/distilled water (3 : 1 : 6 v/v/v).

### 2.5. Enzyme Assays with Protein Substrates

High grade protein substrates (0.1%, w/v) such as gelatin, hemoglobin, bovine serum albumin (BSA), and ovalbumin, from Sigma (St. Louis, MO, USA), were dissolved in buffer and incubated with gentle agitation for 30 min at room temperature with 10 *µ*g of protein from the extracts. The reactions were stopped by addition of 500 *µ*L of 10% (w/v) trichloroacetic acid. The tubes were kept on ice for 10 min and centrifuged at 10,000 ×g for 10 min. The absorbance of the supernatants was measured at 280 nm. One unit of enzymatic activity (U) was defined as the amount of enzyme required to cause an absorbance increase of 0.1 under standard conditions. Specific activity was defined as U·min^−1^·mg^−1^ protein. The results of each series were expressed as the mean value ± SD.

### 2.6. Effect of Protease Inhibitors, Cations, and Reducing and Surfactant Agents on Protease Activity of* C. ensiformis* Extracts

The types of* C. ensiformis* protease activities were investigated using specific inhibitors for the known protease classes and L-TAME as substrate. Different concentrations of protease inhibitors dissolved in water (iodoacetamide, benzamidine (BZA), and ethylenediaminetetraacetic acid (EDTA)), in dimethyl sulfoxide (phenylmethanesulfonyl fluoride (PMSF) and trans-epoxysuccinyl-leucylamido-(4-guanidino) butane (E-64)), in methanol (pepstatin), or in ethanol (N-tosyl-L-phenylalanine chloromethyl ketone (TPCK) and 1,10-phenanthroline (PHE)) were incubated with 10 *µ*g of protein from the extracts for 30 min at room temperature. The reaction was started by addition of the substrate (0.125 mM L-TAME) at 24°C for 15 min, and the activity was measured as described above. Appropriate controls were carried out in parallel using the same enzyme solutions without inhibitors. Inhibition was expressed as the percentage of the appropriate control activity. All inhibitors were purchased from Sigma.

10 *µ*g of protein from each extract were incubated for 30 min at 24°C with 10 mM of the chlorides of calcium, zinc, manganese, and magnesium before the substrate was added. The reactions were performed as described above. The percentage of inhibition was calculated considering the protease activity in the absence of added ions as 100%. Each assay was performed in triplicate and the results were expressed as the mean value ± SD.

The effect of reducing and surfactant agents on* C. ensiformis* proteolytic activity was studied using *β*-mercaptoethanol and Triton X-100, respectively, at 1, 5, and 10%. About 10 *µ*g of protein from the extracts was preincubated for 30 min at room temperature with the agents and the reactions were triggered as described above. The percentage of inhibition was calculated considering the protease activity in the absence of agents as 100%. Each assay was performed in triplicate and the results were expressed as the mean value ± SD.

## 3. Results and Discussion

### 3.1. Protein Measurement and Peptidase Activity against L-TAME of* C. ensiformis* Extracts

As shown in Table 1,* C. ensiformis* extracts have different protein contents and activities against L-TAME. The seed extract presented one of the smallest protein contents and the lowest activity using L-TAME as substrate. On the other hand, leaf extracts showed the highest protein content compared with seed, stem, and root extracts, especially CE-T, suggesting that Tris buffer was more efficient for extracting proteins from the leaves (Table 1). The amount of protein and protease activity showed slight variation according to the season of the year when the plant was collected, the seed extract or the stem extract being the lowest (data not showed).

The protein concentration of legume seeds normally ranges from 20% to as much as 40% and, in general, is the highest among the plant parts [20]. Exceptionally,* C. ensiformis* leaf extracts showed the highest content of proteins (39.53 to 21.74%) in contrast with other leguminous leaves [21–23]. The high content of protein in the leaves probably justifies their use as a vegetable for human consumption and in forage for animals [3]. Leaf extracts also exhibited the highest proteolytic activity against L-TAME when compared with leaf extracts from other species of legume. Similar results were observed for* Crotalaria spectabilis* extracts [23]. The root system is responsible for the acquisition of resources and secretes proteolytic enzymes engaged in the nitrogen assimilation process that involves proteolysis of soil proteins [24]. Furthermore, a notable example of high active stem protease is bromelain (EC 3.4.22.32), a cysteine protease from pineapples (*Ananas comosus, *Bromeliaceae) [25].

### 3.2. Effects of pH and Temperature on the Enzymatic Activity of* C. ensiformis* Extracts

**Figure 1:**
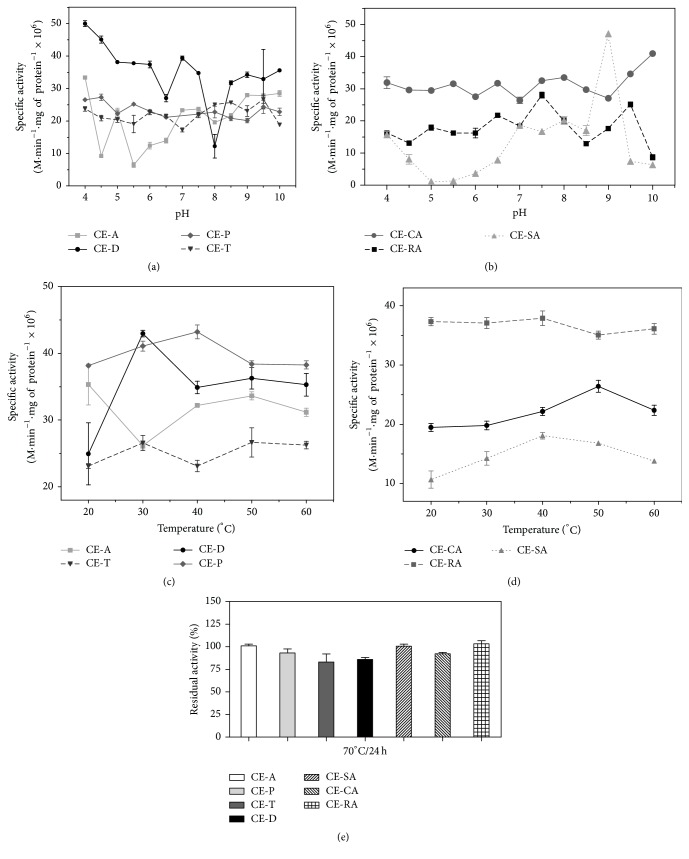
Effect of pH and temperature on peptidase activity of* C. ensiformis *extracts. Ten *μ*g of protein from extracts and the substrate L-TAME (0.125 mM) were used. The following buffers (50 mM) were used: sodium citrate (pH 4.0–6.5), Tris-HCl (pH 7.0–8.5), and sodium bicarbonate (pH 9.0–10). The effects of pH are shown for leaf extract in (a) and for stem, root, and seed extracts are shown in (b), and the effects of temperature on the leaf extract are shown in (c) and for stem, root, and seed extract are shown in (d). For the thermal stability of peptidase activity extracts (10 *μ*g of protein) were preincubated at 70°C up to 24 h without substrate.

The pH influences the enzymatic activity, and determination of the optimum pH is useful to support the protease classification. These pH values also provide clues to the enzymes' location in the cell and their function* in vivo.* Overall, aspartic proteases have a pH optimum in the acidic range, cysteine proteases at a slightly acidic to neutral pH, and serine and metalloproteases in the neutral to alkaline range [26]. The effect of pH on the enzymatic activities of* C. ensiformis* extracts using L-TAME as substrate is shown in Figure 1. All curves exhibit many peaks or inflections that represent increments of activity in different pH ranges, indicating the protease heterogeneity of the extracts. The maximum activity was observed at neutral to alkaline pH ranges for leaf extracts: 7.0 for CE-D, 9.0 for CE-A, and 9.5 for CE-T and CE-P (Figure 1(a)), suggesting the presence of serine and metalloproteases. However, peaks at around 4.0, 5.0, and 6.0 suggested the presence of some aspartic or cysteine protease activities, respectively. Extracts from seeds, root, and stem had the maximal activities in the alkaline pH range: 9.0 for CE-SA and 7.5 and 9.5 for both CE-RA and CE-CA. Secondary peaks between 5.0 and 6.5 were also noticed in these extracts, supporting the idea that the principal peptidase activity is due to serine or metalloproteases, but with some other types of proteases also present. Serine protease activities are the most abundant proteases in all living organisms, including plants as in species of the Leguminosae family [12, 23, 27–29].

The temperature effect on the enzymatic activities was evaluated using different buffers, adjusted to the optimum pH of each extract and temperatures ranging within 20–60°C (Figures 1(c) and 1(d)). Differently from the pH study, the temperature curves did not exhibit pronounced peaks of activity, with exception for CE-D that showed an important peak of maximal activity at 30°C (Figure 1(d)). However, the maximal activities for other extracts were at around 40°C for CE-P, CE-RA, and CE-SA and 50°C for CE-A, CE-T, and CE-CA.

The thermal stability assays were performed incubating* C. ensiformis* extracts for 24 h at 70°C without substrate. Proteases from leaf extracts CE-A and CE-P and seed, stem, and root extracts were the most thermally resistant since the treatment did not affect their activity, while CE-T proteases were the most sensitive, their activities dropping by about 80% (Figure 1(e)).

Temperature influences the velocity of enzymatic reactions by affecting the native structure of proteins and promoting the encounter of the substrate with the active site [30]. Thus, increasing the temperature increases the velocity as long as the native structure of the protein is preserved. Higher temperatures lead to the denaturation of enzymes mainly by breaking hydrogen bonds [31]. Generally, enzymes of* C. ensiformis* extracts continued to show expressive proteolytic activity at high temperatures, with optimal activity at around 40°C and maximal activity at 50°C. Other plant proteases, such as a serine protease from* Artocarpus heterophyllus* latex [32], a serine protease in senescent wheat leaves [33] or the 30 kDa cysteine protease from horse gram (*Macrotyloma uniflorum,* Fabaceae) cotyledons [34], and the aminopeptidase from soybean cotyledons [35], had maximum activity at temperatures similar to those of the proteases from* C. ensiformis *extracts. On the other hand, the 41 kDa serine protease of* C. ensiformis* seeds had an optimum temperature about 60°C [7]. In assays of thermal stability, most of the* C. spectabilis *preserved 100% of activity after incubation at 70°C without substrate. Thus, proteases of these extracts demonstrated very good thermal stability compared with proteases from many other tropical plants since they have the ability to withstand and acclimatize to the environment temperature variations, which are essential to plant survival [28, 36]. The thermal stability of an enzyme is most important for its biotechnological application [31].

### 3.3. Electrophoretic Analysis of* C. ensiformis* Extracts

**Figure 2:**
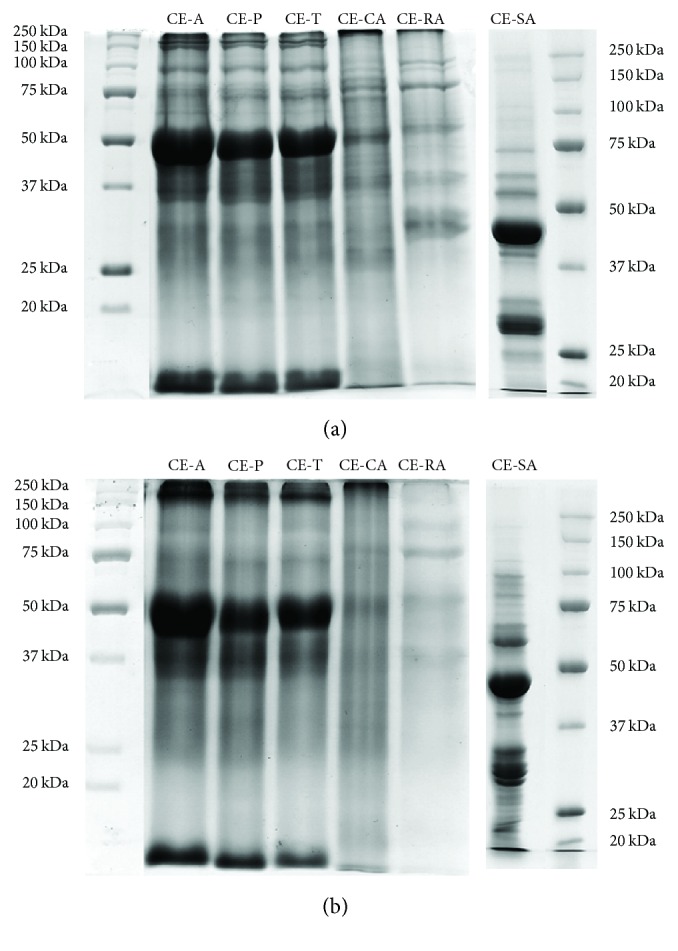
Sodium dodecyl sulfate polyacrylamide gel electrophoresis (SDS-PAGE) of* C. ensiformis *extracts. About 30 *μ*g of protein from each extract was analyzed by SDS-PAGE 12%. (a) Nonreducing and (b) reducing conditions. Standard proteins (*M*_*r*_ in kDa) are on the left and right sides of the gels.

Electrophoretic profiles of the most abundant proteins of each part of* C. ensiformis* were in general similar but presented particular differences (Figure 2). Protein profiles from leaf, root, and stem extracts were very similar under both reducing (Figure 2(a)) and nonreducing conditions (Figure 2(b)), suggesting that these major proteins could be single polypeptide chains. The most pronounced protein of about 50–55 kDa was present in all leaf extracts and other proteins of around 250, 180, 150, 100, 75, and 37 kDa were also observed. It is possible that the 50–55 kDa band contains Rubisco (ribulose-1.5-bisphosphate carboxylase/oxygenase) which is one of the largest and most abundant enzymes in nature. It has a molecular mass of about 560 kDa which consists of subunits with 55 and 15 kDa [37]. Similar protein profiles were also found for other leaf extracts [38]. Electrophoretic profiles of the seed extract changed when *β*-mercaptoethanol was omitted, suggesting the presence of proteins with multiple polypeptide chains. Two proteins with 41 and 28 kDa were observed in both reducing and nonreducing conditions. On the other hand, many proteins of about 160, 110, 90, 85, 80, 75, and 30 kDa that were found under nonreducing conditions were not detected in the presence of a reducing agent.

**Figure 3:**
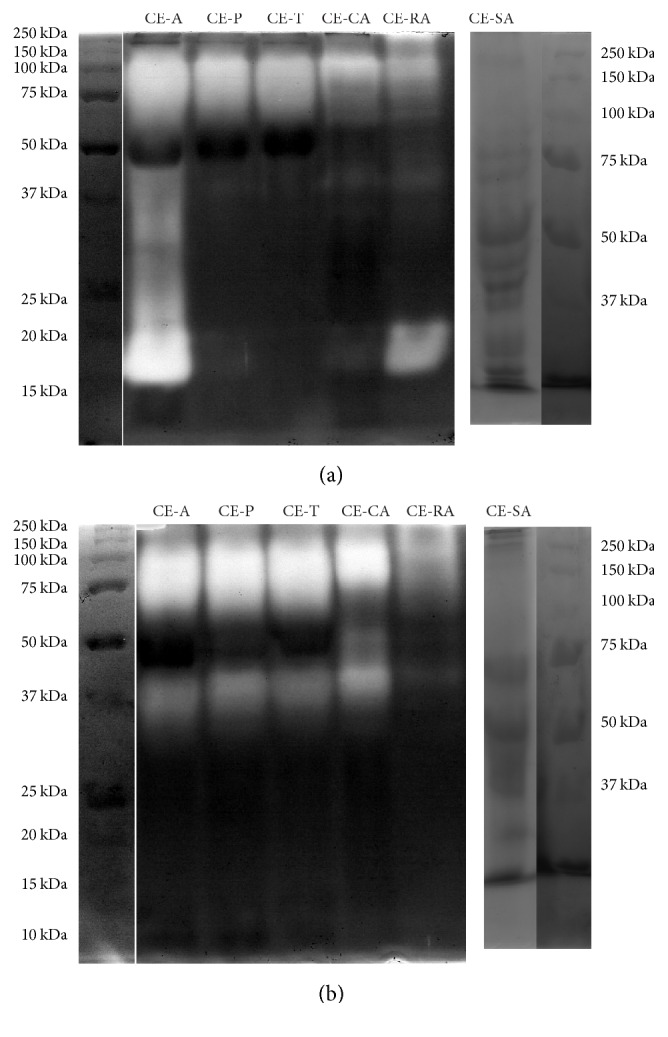
Gelatin substrate-sodium dodecyl sulfate polyacrylamide gel electrophoresis (SDS-PAGE-gelatin) of* C. ensiformis *extracts. The protease activity of about 50 *μ*g of protein from the extracts was analyzed by SDS-PAGE 10%. (a) Nonreducing and (b) reducing conditions. Standard proteins (*M*_*r*_ in kDa) are on the left side of the gels.

The activity of* C. ensiformis* extracts against protein substrates was first evaluated using SDS-PAGE with copolymerized gelatin (Figure 3) and was very useful for studying the proteolytic activity and the molecular masses of the proteases. All extracts, except for the seed extract, showed very important hydrolytic activity against gelatin. The intense proteolysis, under nonreducing conditions, generated a region of activity with about 130–57 kDa, for the leaf extract CE-A, and about 130–60 for other extracts. Besides, CE-A exhibited a strong proteolysis with about 20–15 kDa. Root and stem extracts also showed an additional protease activity in similar region, about 19–16 kDa, which was more pronounced for root extracts. Additional proteolysis can be observed at around 32 and 23 kDa for CE-A and around 40 kDa for root and stem extracts (Figure 3(a)). Under reducing conditions, regions of proteolysis in the region of 140–57 kDa for leaf extracts and 200–84 kDa for CE-CA and 130 kDa for CE-RA were also observed. The 40–37 kDa proteases present in leaf, stem, and root extracts were only demonstrated under reducing conditions and would be derived from higher molecular weight enzymes.

### 3.4. Proteolytic Activity of* C. ensiformis* Extracts

The hydrolytic activity against L-TAME (Table 1) and proteolytic activity against gelatin (Figure 3) were also observed when they were assayed using other proteins such as hemoglobin, BSA, and casein (Table 2). Excepting leaf extracts CE-T and CE-P that failed to show activity against hemoglobin, all extracts hydrolyzed in different ways all protein substrates and the most active were CE-A and CE-D from leaf and stem extract CE-CA. The seed extract did not demonstrate activity against gelatin and had the lowest activity with L-TAME; however, it hydrolyzed all protein substrates especially hemoglobin.

*C. ensiformis *proteases are versatile enzymes because they are able to hydrolyze both peptidomimetic and protein substrates. They cleaved gelatin, hemoglobin, BSA, and casein differentially according to the part of the plant, suggesting that each part has a particular group of peptidases that are responsible for specific functions in the plant's physiology and can be employed for different biotechnological purposes. All* C. ensiformis* peptidases previously reported from seeds are also endopeptidases and thus hydrolyzed protein substrates [27, 39, 40].

### 3.5. Effect of Protease Inhibitors, Cations, and Chemical Compounds on Protease Activity of* C. ensiformis* Extracts

The effects of specific inhibitors on protease activity provide the most reliable information about the catalytic mechanism [19]. Many substances were able to inhibit or modulate the hydrolytic activity of* C. ensiformis* extracts towards L-TAME hydrolysis (Table 3). Phenylmethylsulfonyl fluoride (PMSF), a general serine protease inhibitor, N-tosyl-L-phenylalanine-chloromethyl ketone (TPCK), a chymotrypsin-like serine protease inhibitor, and benzamidine (BZA), a trypsin-like serine protease inhibitor that binds Asp residue of the catalytic triad of serine proteases [41], inhibited the enzymatic activity of leaf extracts in different levels. The most pronounced effect was obtained using BZA that inhibited about 78, 66, and 60% of CE-A, CE-P, and CE-T, respectively, but did not affect CE-D, whereas the most pronounced inhibition of TPCK was about 56% observed for CE-P. Besides, important inhibition of about 71 to 40% by metalloprotease inhibitors EDTA and 1,10-phenanthroline (PHE) was also observed for all leaf extracts. Cysteine and aspartic protease inhibitors, E-64, and pepstatin, respectively, showed little effect on leaf protease activities, suggesting the presence of serine and metalloproteases in* C. ensiformis* leaf extracts. The hydrolytic activity of root extract was completely abolished by EDTA and PHE but was not affected by a specific serine protease inhibitor. However, iodoacetamide reduced about 40% the protease activity of this extract. This amide is an alkylating reagent that binds irreversibly cysteine residues in the active site of enzymes and can inhibit specifically cysteine proteases and nonspecifically serine protease activities [42]. Hydrolytic activity of stem extract was reduced by serine and metalloprotease inhibitors and the most pronounced effect was obtained with BZA. On the other hand, the activity of seed extract was expressively inhibited by TPCK (85%) and the aspartic protease inhibitor pepstatin (91%), suggesting the presence of serine and aspartic protease activities in this extract. Serine and metalloproteases are the most important proteases in* C. ensiformis *extracts as suggested by the assays of the pH influence on the enzymatic activity (Figure 1). Serine proteases are the largest class of proteolytic enzymes in plants, with more than 200 members, displaying functions in the entire life history of plants and are present in all organs, cells, and cellular compartments [1–4].* C. ensiformis *organs, particularly leaves, seeds, and stem, showed important inhibition with serine protease inhibitors. Leaves appeared to have both trypsin and chymotrypsin-like serine protease activity, while seed had only chymotrypsin activity and stem had only trypsin-like proteases. The differences of serine protease expression in plant organs certainly have physiological and ecological importance for* C. ensiformis. *This relevant activity could represent a crucial role in plant defense, since these enzymes interact with phytopathogenic microorganisms and insects [6]. Furthermore, these enzymes are also involved in organ development [43].

All* C. ensiformis *extracts exhibited important metalloprotease activity, with the exception of the seed extract. Plants express zinc and calcium-dependent matrix metalloendopeptidases. However, their exact roles are not yet fully understood but possibly could be involved in remodeling of the extracellular matrix during growth and development, germination, and senescence and in response to biotic and abiotic stresses [44].

Only low inhibition by cysteine protease inhibitors was observed in* C. ensiformis *leaf and stem extracts. The 37 kDa cysteine protease was purified about five thousand times from seeds [6] and it is possible that the experimental conditions employed here were not sensitive to detect the activity of cysteine protease since this enzyme was concentrated with purification. The remarkable inhibition of seed extract activity observed with pepstatin indicated expressive presence of aspartic protease. In plants, these are pepsin-like enzymes involved in constitutive disease resistance and cell survival [5].

The presence of ions can modulate the activity of many proteases. In this study, the peptidase activities of* C. ensiformis* were differently modulated by divalent cations such as Ca^2+^, Zn^2+^, Mg^2+^, and Mn^2+^ (Table 4). They had both positive and negative effect on protease activity except for zinc which enhanced the activity of all extracts. It was possible with zinc to enhance the hydrolytic activities up to 12 times (CE-D) and it was the best activator. Ca^2+^ modulated negatively the activity of CE-A and CE-P and abolished completely the activity of CE-SA. On the other hand, calcium activated the peptidase activity of CE-D, CE-T, and CE-RA. In general, Mg^2+^ was a negative modulator of* C. ensiformis* protease activity, since it decreased the activity of CE-A, CE-D, CE-P, and CE-SA and completely abolished the activity of CE-CA. However, it modulated positively the activity of CE-T about 2 times. Finally, Mn^2+^ decreased the activity of CE-P and completely inhibited the activity of CE-T, CE-CA, and CE-SA, although it activated CE-RA and especially CE-A and CE-D. As metalloprotease activity was detected in all extracts, except in seeds, it was expected that thehydrolytic activity would be modulated by cations. Plant metalloproteases are normally zinc and calcium-dependent enzymes [45] and calcium is known to interfere with the tridimensional structure of enzymes and therefore with their catalytic properties [46].

**Figure 4:**
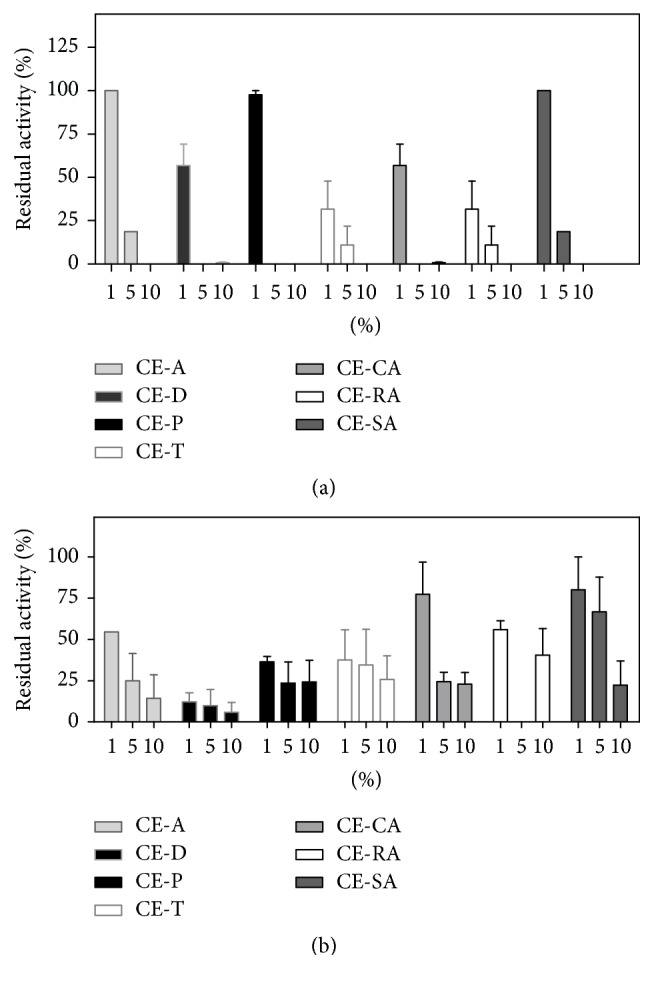
Effects of Triton X-100 (a) and *β*-mercaptoethanol (b) on the peptidase activity of* C. ensiformis* extracts. Extracts (10 *μ*g of protein) were preincubated with agents at the indicated concentration, for 15 min at room temperature. Values are the residual activity of extracts as a percentage of the activity on L-TAME without additives and represent the average of 3 separate experiments carried out in duplicate. The percentage of inhibition was calculated considering the protease activity in the absence of agents as 100%. Each assay was performed in triplicate and the results were expressed as the mean value ± SD.

The influence of reducing and surfactant agents on the stability of peptidase activity was studied using increasing concentrations (1 to 10%) of *β*-mercaptoethanol and Triton X-100. Both agents affected the activities of* C. ensiformis* extracts in different ways (Figure 4). Surfactant Triton at 1% did not affect the activity of CE-A, CE-P, CE-CA, and CE-RA, but reduced the activity of CE-D, CE-T, and CE-SA about 43, 60, and 36%, respectively. Triton at 5% abolished the activity of CE-D and CE-P and reduced substantially the activities of other extracts, and at 10% no protease activity was observed (Figure 4(a)). The activity of* C. ensiformis* extracts seemed to be more resistant to reducing agents because they were active at the higher concentration of *β*-mercaptoethanol (Figure 4(b)). The most resistant extracts included the leaf CE-A and CE-T and stem and seed extracts. Surfactants and reducing agents are part of many protein preparations for biotechnological, pharmacological, or industrial purposes and can affect the activity of certain enzymes [47].

## 4. Conclusion

The present study reported for the first time the protease activity in leaves, stems, and roots of* C. ensiformis, *although proteases have already been described in seeds. All organs of* C. ensiformis *expressed different classes of proteases, showing distinct biochemical and kinetic properties, but the most pronounced protease activity was observed in the neutral to alkaline pH range due to serine and metalloprotease classes, modulated by cations, particularly positively modulated by zinc. All peptidase activity showed remarkable stability in high temperature and good activity in the presence of reducing and surfactant agents, suggesting high potential for biotechnological application.

## Figures and Tables

**Table 1:** Protein quantification and peptidase activity against L-TAME of *C. ensiformis* extracts.

Extract	Protein content (%)^*∗*^	Specific activity [Mol·min^−1^·mg^−1^ protein]
CE-A	26.85	29.34
CE-D	21.74	28.63
CE-P	27.84	21.76
CE-T	39.53	18.83
CE-CA	6.10	34.23
CE-RA	19.05	26.41
CE-SA	11.39	6.36

^*∗*^The amount of protein by mg of extract.

**Table 2:** Proteolytic activity of *C. ensiformis *extracts on protein substrates^a^.

Extract	Proteolytic activity (U/mg)		
	Hemoglobin	Casein	BSA
CE-A	7.3 ± 0.9	7.0 ± 0.4	6.2 ± 0.1
CE-D	9.3 ± 1.6	9.7 ± 0.6	8.4 ± 1.1
CE-P	0.0 ± 0.0	5.6 ± 0.9	1.0 ± 0.7
CE-T	0.0 ± 0.0	5.9 ± 0.3	2.1 ± 0.4
CE-CA	6.8 ± 0.6	6.4 ± 0.6	7.9 ± 1.1
CE-RA	4.0 ± 0.8	3.7 ± 0.8	3.3 ± 1.1
CE-SA	8.1 ± 0.0	1.6 ± 1.7	4.5 ± 1.2

^a^The extracts were incubated with BSA, casein, or hemoglobin for 30 min, reactions were stopped, and absorbance of supernatants was measured at 280 nm. One unit of enzymatic activity (U) refers to the amount of enzyme required to cause an absorbance increase of 0.1 under standard conditions and the specific activity was defined as U·min^−1^·mg^−1^ protein.

**Table 3:** Effects of different types of protease inhibitors on *C. ensiformis* extracts peptidase activity^a,b^.

Target protease	Residual activity (%)							
	Serine			Serine/cysteine	Cysteine	Aspartic	Metalloprotease	

Extract	Inhibitor							
	TPCK (100 *μ*M)	BZA (10 *μ*M)	PMSF (1 mM)	Iodo-acetamide (100 *μ*M)	E-64 (10 *μ*M)	Pepstatin (10 *μ*M)	EDTA (10 mM)	PHE (10 mM)

CE-A	81.14 ± 13.7	21.22 ± 0.4	100.00 ± 0.0	100.00 ± 0.0	88.89 ± 5.0	99.70 ± 0.8	43.57 ± 5.7	58.33 ± 2.1
CE-D	71.7 ± 4.6	100.00 ± 0.0	100.00 ± 0.0	100.00 ± 0.0	94.90 ± 4.0	100.00 ± 0.0	100.00 ± 0.0	44.30 ± 8.6
CE-P	44.0 ± 2.8	34.45 ± 2.4	100.00 ± 0.5	100.00 ± 0.0	91.66 ± 0.7	87.50 ± 9.8	49.15 ± 6.4	66.29 ± 3.3
CE-T	100.0 ± 0.0	40.09 ± 3.5	93.08 ± 0.4	100.00 ± 0.0	82.88 ± 3.8	78.70 ± 0.3	79.22 ± 1.9	28.57 ± 0.0
CE-CA	79.8 ± 1.2	14.60 ± 0.9	97.34 ± 2.9	98.82 ± 1.2	97.11 ± 8.0	90.86 ± 5.8	49.64 ± 2.5	42.14 ± 9.2
CE-RA	95.3 ± 13.6	100.00 ± 2.8	100.00 ± 2.5	59.00 ± 7.3	100.00 ± 2.1	91.00 ± 0.0	38.00 ± 0.8	0.00 ± 0.0
CE-SA	14.5 ± 21.8	100.00 ± 0.0	100.00 ± 0.0	100.00 ± 0.0	100.00 ± 0.0	8.90 ± 4.5	100.00 ± 0.0	100.00 ± 0.0

^a^The extracts were preincubated with each reagent for 30 min at room temperature. The remaining activity was assayed by incubation with 0.125 mM L-TAME. ^b^Values are the remaining activity of serine protease as percentage of the activity on L-TAME without compounds, measured as described in “Materials and Methods,” and represent the average of 3 separate experiments carried out in duplicate. BZA: benzamidine; E-64: L-*trans*-epoxysuccinyl-leucylamido-(4-guanidino) butane; EDTA: ethylenediaminetetraacetic acid; PHE: 1,10-phenanthroline; PMSF: phenylmethanesulfonyl fluoride; TPCK: *N*-tosyl-L-phenylalanine chloromethyl ketone.

**Table 4:** Effects of cations on *C. ensiformis* extracts peptidase activity^a,b^.

Residual activity (%)^a^				

Extract	Cation			
	Mg^2+^	Mn^2+^	Zn^2+^	Ca^2+^

CE-A	63.4 ± 13.7	758.8 ± 2.1	854.0 ± 126.9	25.2 ± 10.4
CE-D	41.8 ± 9.3	1010.5 ± 189.6	1276.9 ± 9.4	187.98 ± 23.4
CE-P	41.3 ± 19.9	36.5 ± 32.6	275.7 ± 23.8	70.4 ± 5.6
CE-T	226.9 ± 14.4	0.0 ± 0.0	328.8 ± 40.8	274.4 ± 37.0
CE-CA	0.0 ± 0.0	0.0 ± 0.0	782.4 ± 8.3	106.3 ± 7.4
CE-RA	105.4 ± 8.3	167.7 ± 2.8	144.2 ± 26.4	121.5 ± 10.6
CE-SA	68.7 ± 8.8	0.0 ± 0.0	263.0 ± 104.8	0.0 ± 0.0

^a^The extracts were preincubated with each cation for 30 min at room temperature. The remaining activity was assayed by incubation with 0.125 mM L-TAME. ^b^Values are the remaining activity of serine protease as percentage of the activity on L-TAME without compounds, measured as described in “Materials and Methods,” and represent the average of 3 separate experiments carried out in duplicate.

## Abbreviations

BSA: Bovine serum albumin
BZA: Benzamidine
CE: * Canavalia ensiformis*
CE-A: Aqueous leaf extract from CE
CE-CA: Aqueous stem extract from CE
CE-D: Detergent leaf extract from CE
CE-P: Phosphate leaf extract from CE
CE-SA: Aqueous seed extract from CE
CE-RA: Aqueous root extract from CE
CE-T: Tris leaf extract from CE
E-64: L-*trans*-Epoxysuccinyl-leucylamido-(4-guanidino) butane
EC: Enzyme classification
EDTA: Ethylenediaminetetraacetic acid
PHE: 1,10-Phenanthroline
PMSF: Phenylmethanesulfonyl fluoride
SDS-PAGE: Sodium dodecyl sulfate polyacrylamide gel electrophoresis
L-TAME: *N*-*α*-Tosyl-L-arginine methyl ester
TPCK: *N*-Tosyl-L-phenylalanine chloromethyl ketone
U: Enzymatic activity.
