# Corrigendum to “Proteases from* Canavalia ensiformis*: Active and Thermostable Enzymes with Potential of Application in Biotechnology”

**DOI:** 10.1155/2016/9872540

**Published:** 2016-10-12

**Authors:** Rayane Natashe Gonçalves, Suellen Duarte Gozzini Barbosa, Raquel Elisa da Silva-López

**Affiliations:** Department of Natural Products, Farmanguinhos, Oswaldo Cruz Institute (FIOCRUZ), Avenida Brasil 4365, 21045-900 Rio de Janeiro, RJ, Brazil

In the article titled “Proteases from* Canavalia ensiformis*: Active and Thermostable Enzymes with Potential of Application in Biotechnology” [1], the name of the first author was given incorrectly as Rayane Natshe Gonçalves. The author's name should have been written as Rayane Natashe Gonçalves. The revised authors' list is shown above.
